# Chatbots for HIV Prevention and Care: a Narrative Review

**DOI:** 10.1007/s11904-023-00681-x

**Published:** 2023-11-27

**Authors:** Alastair van Heerden, Shannon Bosman, Dallas Swendeman, Warren Scott Comulada

**Affiliations:** 1 Center for Community Based Research, Human Sciences Research Council, Old Bus Depot, Pietermaritzburg, 3201 South Africa; 2 SAMRC/WITS Developmental Pathways for Health Research Unit, Department of Paediatrics, School of Clinical Medicine, Faculty of Health Sciences, University of the Witwatersrand, Johannesburg, Gauteng South Africa; 3 Department of Psychiatry and Biobehavioral Sciences, David Geffen School of Medicine, University of California, Los Angeles, CA USA; 4 Center for Community Health, Semel Institute for Neuroscience and Human Behavior, University of California, Los Angeles, CA USA; 5 Department of Health Policy and Management, Fielding School of Public Health, University of California, Los Angeles, CA USA

**Keywords:** HIV prevention, Chatbots, Conversational agents, Large language models, Generative AI

## Abstract

**Purpose of Review:**

To explore the intersection of chatbots and HIV prevention and care. Current applications of chatbots in HIV services, the challenges faced, recent advancements, and future research directions are presented and discussed.

**Recent Findings:**

Chatbots facilitate sensitive discussions about HIV thereby promoting prevention and care strategies. Trustworthiness and accuracy of information were identified as primary factors influencing user engagement with chatbots. Additionally, the integration of AI-driven models that process and generate human-like text into chatbots poses both breakthroughs and challenges in terms of privacy, bias, resources, and ethical issues.

**Summary:**

Chatbots in HIV prevention and care show potential; however, significant work remains in addressing associated ethical and practical concerns. The integration of large language models into chatbots is a promising future direction for their effective deployment in HIV services. Encouraging future research, collaboration among stakeholders, and bold innovative thinking will be pivotal in harnessing the full potential of chatbot interventions.

## Introduction

Chatbots, also known as conversational agents, have gained significant attention in various fields, including education, commerce, entertainment, and healthcare [1]. These systems, which communicate with users using natural language, mimic human behaviour and are often connected to messaging services, web pages, and mobile applications [2]. They can be classified into two types: rule-based and AI-based [3••, 4•]. Rule-based chatbots use a state machine and pre-defined question-and-answer structures to control conversations. On the other hand, AI-based chatbots utilize AI techniques for natural language understanding (NLU) and natural language generation (NLG), allowing them to understand natural language, maintain different conversation contexts, and generate fluid and coherent responses [2, 5]. Building on the existing body of knowledge, this review uniquely synthesizes recent developments, advancements, and challenges in the application of chatbots and large language models (LLMs) in HIV prevention and care, along with providing an insightful perspective on future directions, thereby offering a comprehensive understanding of this rapidly evolving intersection of technology and healthcare.

## Approach

For this review, Google Scholar, Semantic Scholar, PubMed, and arXiv were carefully searched using keyword combination phrases such as “chatbots and HIV prevention”, “large language models and HIV prevention”, and “AI and HIV Care” to amass studies relevant to the intersection of chatbots and HIV prevention and care. Ideas were also developed through conversations with peers working in the field. The principal criterion for the inclusion of studies was their relevance to the topic, with a primary focus on English literature published within the past 5 years to ensure the recency and pertinence of the findings. The review aimed to provide a qualitative synthesis of recent literature on the topic. Unlike systematic reviews, this review adopted a more interpretive, discursive approach, allowing for the incorporation of a broader range of materials and the elucidation of trends, themes, and emerging insights in the field of study. It provided the opportunity to explore the evolving narrative of chatbots in HIV prevention and care, surfacing the multifaceted ways in which conversational agents have been leveraged to address the challenges inherent in HIV prevention efforts and the provision of care.

## HIV Prevention and Care

The use of conversational agents (chatbots) in HIV prevention is still in its infancy. Nonetheless, there has been significant investment in their development and use, including in healthcare. As evident in Table 1, chatbots have been posited as a promising tool to provide personalized health information and offer emotional support in diverse and resource-constrained contexts [6••, 7, 8•]. Notably, a systematic review of chatbot interventions in Africa found that 4 (33%) of the 12 papers meeting inclusion criteria focused on the use of chatbots in HIV prevention and care. Beyond the finding of how few papers exist on the topic in Africa, other insights from the review include infrastructure challenges (mobile phones, data costs, and electricity), the barrier of indigenous language use, a focus on user experience and design, and regulatory, ethical, and data security issues [6••]. A broader review of chatbots in healthcare reports shows that the chatbot evidence base consists primarily of descriptive, quasi-experimental, and qualitative studies. Most studies are aimed at treatment, rather than prevention, with monitoring and health care system support being prevalent. Chatbots were found to typically be delivered by SMS or app and almost always using text rather than audio, video, virtual reality, or other modalities [3••]. A third review of mHealth tools to promote HIV pre-exposure prophylaxis (PrEP) uptake and adherence found that chatbots, primarily rules-based rather than AI to date, were included among the most common mHealth interventions encouraging HIV prevention that also included gamification, medication logs, testing location maps, and notification reminders [8•].

**Table 1:** Summary of literature presented on chatbot use in HIV care and prevention

Chatbot name	Delivery method	Primary focus	Target population	Notable findings/benefits
General chatbots (e.g. askNivi)	SMS or app	Treatment/prevention	General population	Most are text-based, and some focus on monitoring and healthcare system support
Chatbots for TGW (e.g. Amanda Selfie)	Facebook Messenger	Prevention of HIV infection and promotion of PrEP	Adolescents (transgender focus)	Well accepted as a peer educator, providing information on sexual health and gender identity issues, identifying ATGW at a greater risk of HIV infection, offering access to PrEP clinics, and improving linkage and retention to PrEP care
Chatbots for MSM	SMS, Facebook Messenger, web platforms, and mobile apps	Provide information, education, counselling, and support on topics such as HIV prevention, sexual health, PrEP, and mental health	Men who have sex with men (MSM)	Potential to reach a large and diverse audience, offering personalized and confidential interactions, improving access to health care services and resources, and enhancing user engagement and satisfaction
Chatbots for FSW	Prototype	Qualitative discussion with FSW about chatbots	Cis-gender female sex workers	Openness to chatbots. Barriers include inconsistent phone ownership and privacy concerns
Chatbots for testing (e.g. Nolwazi bot)	Telegram	HIV self-testing support	General population (isiZulu-speaking)	High acceptability, especially among men. Eighty percent preferred chatbot experience over human interaction
Chatbots for health care workers	Virtual focus groups	Improve service capacity and quality	HIV research staff, intervention coaches, and community advisory boards	Chatbots automate check-ins, follow-ups, and referrals, offering personalized support and reducing staff burden. Most studies focus on text-based chatbots in high-income countries, limiting generalizability

### Key Populations

Several chatbots, such as the pioneering “Amanda Selfie” in Brazil, have been developed with a focus on key populations. Amanda Selfie, a transgender chatbot used to stimulate PrEP interest in adolescents, can facilitate sensitive discussions on sex, STIs, and PrEP, and even identify individuals at higher risk for HIV [9]. Using the theoretical framework of Davis [10] qualitative work with men who have sex with men (MSM) in Malaysia found that the perceived usefulness of chatbots hinged around questions of whether the information provided by the chatbot was accurate, and whether or not it could provide emotional support. Ease of use was influenced by considerations of cost, convenience of access, and software bugs [11]. Similar results were obtained from focus group discussions with cis-gender female sex workers in South Africa who were found to be open to chatbots and other mHealth tools for HIV care. Additional barriers to accessing and using these tools emerge in low-income groups such as these women who note that inconsistent phone ownership and threats to the privacy of such tools dues to shared phone ownership [12]. Finally, the Nolwazi bot, an isiZulu-speaking conversational agent for HIV self-testing support, showed high acceptability among users, particularly men; around 80% of those engaging with the chatbot preferred the experience over talking with a human counsellor and 77% reported that they felt they were talking with a real person [13, 14]. A key benefit noted across many of these studies is the widely reported perception that chatbots are less “judgmental” compared to interacting with a person, making people more likely to share sensitive information with them [7]. Engaging directly with chatbots can also improve engagement in HIV testing and prevention for high-risk groups who may face barriers to receiving care at traditional primary healthcare facilities by providing access to curated information, increasing anonymity and reducing stigma [15].

### HIV Prevention and Care Workforce

It is not only users who express interest in the application of chatbots to HIV prevention and care. Recent group interviews with HIV research assessment staff, intervention coaches, and community advisory board members uncovered interest in the use of chatbots to improve capacity, consistency, convenience, and quality of services, for example, by automatically conducting check-ins, follow-ups, referral linkages, and scheduling for their clients [16]. The integration of chatbots into a diverse range of health settings allows for personalized and accessible support, including the provision of appropriate resources and treatment recommendations based on patient responses, thereby reducing staff burden. However, the current body of research is not representative of diverse geographies, cultures, and age groups, with most studies conducted in high-income countries and focusing on the use of text-based chatbots [3••, 6••]. This lack of diversity limits the generalizability of the findings.

## Current Limitations

**Fig. 1:**
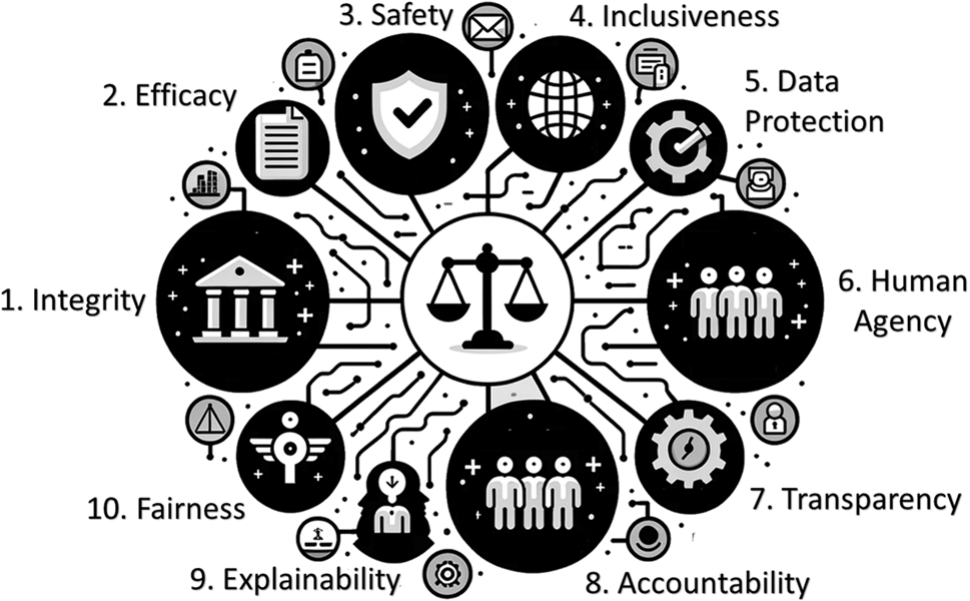
10 principals of the RESET Framework [18]

The use of LLM or AI chatbots in HIV prevention and care raises safety, ethical, and practical concerns, particularly when it comes to providing medical advice. Rule-based chatbots are much more controlled in terms of content responses, but programming permutations in dialogue flows can become unwieldy when attempting to anticipate all possible user responses and when covering diverse topics and actions. For AI chatbots, privacy rights, confidentiality, the potential to provide biased and fabricated responses (i.e. “hallucinate”), data protection, patient engagement, costs, and staffing requirements are among the current issues that need to be addressed before chatbots become more useful and widely implemented in the field of HIV prevention [12, 15]. This is even more relevant in the aftermath of the COVID-19 pandemic accelerating the adoption of telehealth, mHealth, and chatbots in public health and healthcare [12, 15, 17]. Governance frameworks and principals, such as RESET, a set of 10 AI chatbot healthcare ethics principals (Fig. 1) encompassing topics noted above, are helpful in assisting careful consideration of the multiple challenges of ethically deploying a healthcare AI chatbot, but the proliferation of such advice is still fragmented and in some cases contradictory [18]. Alongside these guidelines, calls have been made to establish standards of comparative criteria to evaluate and validate chatbot deployments and safeguarding frameworks for user protection in public health domains [7].

Current chatbots that leverage LLMs to provide a more conversational tone require significant computational resources and energy for training, raising concerns about their environmental impact [19]. There are also concerns that the speed at which adoption of these models has occurred has left many open questions about their limitations and the potential for developing emergent capabilities that are unknown and potentially misaligned with the development team’s goals. Technical intricacies such as prompt brittleness, the inability to do simple reverse logic, e.g. A is the mother of which famous person B (can answer), who is famous person B’s mother? (unable to answer), and outdated knowledge impacts the effective utilization of these models and highlights the need for further exploration and guidelines for their use [20]. Moreover, neural models trained on large datasets can replicate and amplify negative, stereotypical, and derogatory associations in the data. Worse, inappropriate responses from conversational AI systems in emergency situations can have severe consequences, including potential life-threatening outcomes. The integration of LLMs into chatbots is also hampered by current context window limitations which effectively give chatbots very short memories and make continuity over multi-turn interactions (i.e. more than one conversation session) difficult. This may only be a short-term challenge as multiple efforts are underway to address this challenge.

From the user’s perspective, trustworthiness and accuracy of information are both important factors in people’s abandonment of consultations with diagnostic chatbots [7]. This concern is particularly noteworthy in current LLM chatbots that sometimes suffer from “hallucination” by favouring sentence coherence over factual accuracy. Co-design approaches that incorporate insights from users and health professionals have been proposed to combat some of the complexity inherent in designing chatbots. Despite these concerns, the potential of AI in transforming healthcare is undeniable. As regulatory bodies work to implement mechanisms to address AI as a medical device, and as trust, clinical safety, and reliability are prioritized, the future of AI in healthcare looks promising.

## Recent Advancements and Future Directions

The advent of artificial intelligence (AI) has brought about significant transformations in various sectors, including HIV research. Sundar Pichai, CEO of Google, posits that AI will have a more profound impact on humanity than fire, electricity, and the internet [21]. The transformative potential of artificial intelligence (AI), particularly generative AI models like GPT, in healthcare is increasingly being recognized. LLMs, such as ChatGPT and Claude, have shown remarkable ability to comprehend and generate human-like text, leveraging medical data and knowledge to transform clinical decision support, patient communication, and data management in healthcare [22]. In the realm of medical education, LLMs have also shown promise, aiding learning and problem-solving, and demonstrating proficiency in medical knowledge without specialized training [22, 23]. Generative AI language models are also beginning to be applied in clinical decision support (CDS). Recent results from a CDS study analyzed decision support messages generated by ChatGPT (not the newer GPT-4; 36 suggestions) and humans (29 suggestions) for 7 alerts. Of the 20 suggestions that on review by an expert panel were ranked highest, 9 were generated by ChatGPT [24]. An HIV prevention chatbot that can aid in prevention messaging, encourage self-testing, and personalized treatment strategies would be transformational in a low-resource setting. They would have the potential to reach many individuals, especially those who may face barriers to accessing traditional healthcare services.

Future work should rigorously address the optimization of chatbot design and functionality to ensure maximal utility, safety, and user-friendliness (Table 2). This encompasses not only the aesthetic and interactive aspects but also the underlying LLMs that will certainly underpin the next generation of chatbots. Fine-tuning models to allow for better understanding and response to user inputs, particularly in a medical context, is crucial. Furthermore, the inclusion of features that enhance accessibility, such as voice recognition and multilingual support, can significantly broaden the reach and applicability of these interventions. The advancement of AI-driven chatbots in HIV prevention and care necessitates concerted collaborative efforts between healthcare providers, researchers, developers, and end-users. Through collaborative efforts, a thorough understanding of the needs, preferences, and barriers faced by the target populations can be garnered. Moreover, this collaboration can facilitate the translation of research findings into actionable interventions, thus bridging the gap between theory and practice. The potential of chatbots to transform HIV prevention and care in low-resource settings hinges on the scalability and sustainability of these interventions. Exploring innovative funding models, establishing partnerships with local health departments, and leveraging existing healthcare infrastructures are potential strategies to ensure the long-term viability and scalability of chatbot interventions.

**Table 2:** Summary of the benefits, challenges, and insights gathered from the extensive text on the use of chatbots in HIV prevention and care

Benefits	Challenges	Insights Gathered
-Personalized health information -Promising tool in diverse and resource-constrained contexts -Can facilitate sensitive discussions (e.g. “Amanda Selfie”) -Perceived as less “judgmental” compared to human interactions -May increase anonymity and reduce stigma -Provides access to curated information -Promises to transform healthcare paradigms and democratize access -Scalability and sustainability potential in low-resource settings	-Infrastructure challenges: mobile phones, data costs, electricity -Indigenous language use barrier -Regulatory, ethical, and data security issues -Concerns about chatbot accuracy and “hallucinations” -Privacy concerns, especially with shared phone ownership -Environmental concerns due to high computational resource usage -Trustworthiness and accuracy concerns from users -Limited research in diverse geographies, cultures, and age groups	The current chatbot evidence base consists mainly of descriptive, quasi-experimental, and qualitative studies -Concerns about biased and inappropriate responses -Potential for misaligned emergent capabilities -Collaboration between stakeholders is crucial for effective chatbot development -AI advancements in healthcare require rigorous design and functionality optimization -Chatbots in healthcare have potential disruptive capabilities

While integration of chatbots into existing health systems and in support of health care providers are called for, this moment also asks of us the careful consideration about whether we want the future of HIV care to simply be the current system with chatbots in support of it or whether the future could and should look radically different as LLMs move towards artificial general intelligence (AGI). Healthcare is a bureaucratic fortress brimming with vested interests and a lucrative revenue model. While regulation and oversight are clearly important to protect people from harm, it may be that these instruments become used by powerful stakeholders to steer away from the disruption of existing hierarchies. Chatbots harnessing LLMs hold the potential to dismantle longstanding barriers to healthcare, ushering in an era of unprecedented accessibility and personalized support, particularly in resource-limited settings. A balanced and careful path forward is imperative, one that navigates ethical and safety concerns while keeping the transformative potential alive. Ongoing dialogue should move beyond the simplistic narrative of chatbots merely complementing human providers and venture into the truly disruptive nature of this innovation, exploring how chatbots could redefine healthcare paradigms, democratize access, and potentially dismantle existing power structures that often act as gatekeepers to quality care. To realize this future requires bold and innovative thinking alongside pilot testing, implementation science, and large-scale randomized controlled trials (RCTs) that test the full capabilities of HIV chatbot interventions, especially ones that incorporate the technical advances made in the last 18 months.
